# Beyond pilotitis: taking digital health interventions to the national level in China and Uganda

**DOI:** 10.1186/s12992-017-0275-z

**Published:** 2017-07-31

**Authors:** Fei Huang, Sean Blaschke, Henry Lucas

**Affiliations:** 1 National Center for TB control and prevention, China CDC, Beijing, China; 2 UNICEF, Kampala, Uganda; 3 Institute of Development Studies, Brighton, BN1 9RE UK

**Keywords:** Health management information systems, Digital health, Disease surveillance, Low and middle income countries

## Abstract

**Background:**

Innovation theory has focused on the *adoption* of new products or services by individuals and their market-driven *diffusion* to the population at large. However, major health sector innovations typically emerge from negotiations between diverse stakeholders who compete to impose or at least prioritise their preferred version of that innovation. Thus, while many digital health interventions have succeeded in terms of adoption by a substantial number of providers and patients, they have generally failed to gain the level of *acceptance* required for their integration into national health systems that would promote sustainability and population-wide application. The area of innovation considered here relates to a growing number of success stories that have created considerable enthusiasm among donors, international agencies, and governments for the potential role of ICTs in transforming weak national health information systems in middle and low income countries. This article uses a case study approach to consider the assumptions, institutional as well as technical, underlying this enthusiasm and explores possible ways in which outcomes might be improved.

**Methods:**

Literature review and case study analysis.

**Results:**

The two systems considered have had considerable success in terms of gaining and maintaining government support and addressing the concerns of providers without compromising their core elements. In Uganda, the system has flourished in spite of severe resource constraints, using a participatory approach that has encouraged a high level of community engagement. In China, concern with past failures generated the political will to build a high quality surveillance system, using the latest technology and drawing on a highly skilled human resource base.

**Conclusions:**

Both example stress the importance of recognising the political, social and historical context within which information systems have to function. Implementers need to focus as much on the perceptions, attitudes and needs of stakeholders as on the technology. Implementers should distinguish between factors which may influence engagement at an institutional level and those aimed at supporting and supervising individuals within those institutions. Finally, we would suggest that designing interoperability into systems at the outset, rather than assuming that this can be achieved at some point in the future, may prove far easier in the longer term.

## Introduction

There is a voluminous literature on a multiplicity of digital health innovations in middle and low income countries and in recent years a growing number of evaluation studies that have demonstrated beneficial impacts from these interventions. However, it remains the case that the great majority have been limited in scale and undertaken in stand-alone, vertical project mode. The term ‘pilotitis’ has been used to express the frustration of many of those in the health sector at the continuing emphasis on demonstrating successful outcomes from narrowly focused interventions targeting relatively small populations. The clearest example of such frustration was provided by the actions of the government of Uganda which initiated a moratorium in 2012 [1], demanding that future interventions prioritised interoperability, sustainability and conformity to existing MoH cyber laws and data requirements. In addition, success has often been described from a technical, rather than health outcomes, perspective and costs understated, for example when implementing agencies fail to include pro-bono support provided by their staff members, equipment donations from manufacturers or cost subsidies by telecoms companies.

The aim of this paper is explore the potential for new information and communications technologies (ICTs) to transform *national* health information systems [2]. While there are a number of initiatives underway in this area, it appears to have attracted limited attention in the literature, where discussion is typically limited to technical rather than health systems issues. The paper addresses this objective using case studies of interventions in two countries, China and Uganda, that have designed and implemented ICT-based national reporting systems. We regard the comparison between these examples as of particular interest in that, while both countries have enthusiastically embraced these technologies over recent years, they differ radically in terms of (a) the level of resources which they have been able to dedicate to health information systems and (b) the nature of their healthcare systems.

China is a transition economy that has experienced long-term rapid growth, allowing the allocation of ample financial and human resources to the development of a new information system which has also benefited from strong political support. However, public health facilities in China have a large degree of autonomy, depend mainly on payments either by patients or insurance agencies and operate under “*an archaic and complex governance structure*” (p835) [3], often resulting in what might be seen as a ‘management by exception’ regime, in which responsible authorities will typically only intervene if it becomes apparent that a hospital is failing to meet service provision or financial obligations. Reforms, including those relating to information systems, will typically be introduced by a process of negotiation, not imposition.

The health system in Uganda has a history of innovation in the use of ICT, supported by multiple international donors, but very limited resources even for the provision of basic services. One potential advantage in terms of information systems coverage is that, as set out in the Health Sector Strategic Plan [4], the Ministry of Health (MoH) views both the public sector, where staff are salaried government employees and treatment is, at least in theory, free of change, and the not-for-profit (NFP) sector, which is a major provider in poorer rural areas, as components of an integrated system. District Health Teams are held responsible for the overall performance of services in their district, to the extent that the MoH produces a ‘district league table’ [5] using an HMIS database which is based on a unified reporting system for public and NFP sectors [6, 7].

## Literature overview

There is a long history of failing or at least deficient health information systems in many countries [8], not only in terms of information relating to the management of services but also that on specific population health concerns and general health status. While there have been multiple attempts by both government and international agencies to improve routine data systems in the health sector, there is still a heavy reliance on surveys (for example the Demographic and Health Surveys) or vertical program data systems (for example on AIDS, TB or malaria) when timely, high quality data is required. The recent outbreaks of Ebola and Zika have again highlighted the enormous potential value of reliable, timely surveillance data and the risks associated with its absence [9]. A number of UN agencies (UNICEF, WHO), international donors (DFID, USAID, the World Bank) and agencies (IHME, JHU-GmI, MEASURE Evaluation, PARIS 21) now appear to be convinced that recent developments in ICTs will provide the means to transform health information systems and at last provide policy makers with the data they need to make informed decisions in the health sector that reach the least advantaged.

The starting point for this article is a concern that this enthusiasm is often based on optimistic assumptions about the innovation process (essentially that ‘good’ innovations will inevitably flourish), while the need is to identify specific health systems challenges and assess the extent to which ICTs might play a role in addressing these. Too often the focus is limited to defining an appropriate technical package – reporting formats, equipment, software, training, etc. – which long experience in OECD countries suggests is a necessary but by no means sufficient requirement [10]. Failure may just as easily result from institutional or social constraints, and stakeholder analysis may be as important as traditional information systems analysis [11, 12].

There is also a common assumption, again contrary to experience relating to both public and private sector organisations in highly development economies, that only the documented, formal information system need be considered. In practice this often has limited relevance for the *effective* information system, which can only be understood by detailed observation and analysis (‘Soft Systems Analysis’ [13] is commonly used in the private sector for this purpose). There seems a serious risk that rather than reforming health information systems in ways that encourage improved policy making and hence better health outcomes, ICT innovations may improve some technical aspects of data availability, timeliness or quality, while failing to address inherent weaknesses in the overall system whereby information feeds into the policy making process [14].

A more nuanced understanding of the innovation process relevant to health information systems may enhance the prospects for success. The Oxford English Dictionary defines an ‘innovation’ as a “*new method, idea, product,* etc.”, devised by one or more ‘experts’ which impacts on a given population when adopted by some members of that population. Röling [15] suggests that this view of the innovation process has remained extremely influential, partly because the experts concerned find it very attractive. Much of the early work on innovations [16] focused on innovation *diffusion* from those early adopters to the population at large. Given that much of this work derived from the field of marketing and economics, it is not surprising that the focus was on the role of competition as a driver of diffusion, with successful innovations emerging as a consequence of the rational decisions of millions of individual actors.

On the other hand, health innovations often emerge as a result of a series of complex interactions involving multiple actors including scientists, providers, clients, competitors, regulators and various other private and public organizations [17]. Decisions to adopt are typically made not by isolated individuals but by groups and organisations whose members may have very different perceptions as to the potential costs and benefits of any given innovation. In some cases, as in the examples considered here, innovations may be initially imposed by a policy directive from a government ministry.

In a wide ranging review, Länsisalmi et al. [18] makes the case that such top-down health service innovations can be seen as especially complex and problematic. They point to substantial evidence as to the difficulties of changing the attitudes and practices of healthcare workers, who may perceive that there are risks associated with an innovation, perhaps including that of adversely affecting health outcomes, with potentially serious ethical, professional, social and/or financial implications. Providers, particularly those with high level qualifications and/or many years of experience, typically place a high value on their autonomy and reputation, and may resist innovative practices [19]. They may be particularly unconvinced as to the benefits of innovations, such as information systems, that are outside their area of expertise.

Evidence from a review of eHealth innovations [12] indicates that centralised diffusion systems such as those considered here were more effective in disseminating authoritative evidence and preserving the ‘fidelity’ [20] of interventions, but less successful in terms of adopter engagement, partly because of strict limits on the extent to which implementation could be modified to address the concerns of specific organisations or stakeholders. Even innovations apparently involving purely technical changes, such as the introduction of a new health information system, may have complex socio-political implications in terms of changes in logistics, working practices, organisational arrangements and institutional requirements. Retaining implementation fidelity will often involve a battle of wills, with implementers resisting demands for the introduction of variations in innovation components in line with local circumstances and actors.

This entails a recognition that often “*use and development are twin processes and that they unfold in real time, mutually shaping each other as learning by using expands or narrows the technology’s scope of application*” (p302) [21]. Greenhalgh and Russell [12], argue that so-called ‘evidence based’ health sector innovations are typically subjected to a convoluted series of negotiations between stakeholders which serve to mediate often highly contested understandings and perceptions. Consoli and Mina [21] suggest that innovations implemented to solve a given problem will typically result in a series of new problems necessitating appropriate adaptations. Implementation can thus be viewed as “*trajectories of problems and solutions*” (p309).

Kiwanuka et al. [22], in a study of the District Health Information System in Uganda, which will be discussed further below, emphasises the need to distinguish clearly between the ‘adoption’ of such an innovation by health providers – hard to resist when that innovation is introduced by government – and its ‘acceptance’ by those providers [23]. Following Kollmann [24], they discuss the ‘acceptance process’ which can be seen as a dynamic, often non-linear, progression from initial perceptions of and attitudes to a top-down intervention, through its, possibly reluctant, adoption to its acceptance, if all goes as planned, as a routine activity. This suggests that adoption can be seen as simply the first and perhaps least problematic phase in the drive towards the integration of an innovation into the health system. The greater risk is that of ‘sustainability failure’ [25, 26], where projects either fail completely after a period of apparent success or gradually diverge so far from their original intentions, with increasing ‘design-actuality gaps’ [26], that many of their intended beneficial outcomes are sacrificed. The case studies discussed below may be instructive in that they appear to have avoided, at least to a considerable degree, these twin pitfalls.

## The China case study

The outbreak in late 2002 of what came to be known as severe acute respiratory syndrome (SARS) revealed serious limitations in China’s disease surveillance system. For example, provincial governments had to obtain authorisation from the Ministry of Health (MoH) before announcing an epidemic and the list of mandatory reportable infectious diseases was very narrowly defined, such that local governments were not required by law to report cases of what was diagnosed as atypical pneumonia. As fear of SARS disrupted all aspects of life in China, the impact of the disease rapidly transformed from a major public health concern to a serious social and economic crisis [27]. It elicited a dramatic response from government, including a massive mobilisation of health providers and the establishment of a US$1 billion fund to support SARS prevention and control, which rapidly bought the epidemic to an end by August 2003.

As one component of a major series of reforms initiated in response to failures in addressing the SARS outbreak, the government established the Infectious Disease Reporting Information Management System (IDRIMS). Managed by the Centre for Disease Control and Prevention, this is the world’s largest web-based, real-time surveillance system and is intended to provide a common platform that will enable all health facilities in the country to provide information on 37 notifiable diseases within 24 h of diagnosis [28, 29]. It involved an investment of some US$100 million in hardware, software, programmer salaries and staff training [30]. By standardising epidemic disease surveillance across all health institutions, it is claimed to have improved data timeliness, completeness and validity. Average reporting time is said to have decreased by a factor of 10, the number of complete reports increased by 33% and the number of missing reports substantially reduced. By the end of 2010, it was being used in 68,000 facilities, 97% of county and above hospitals and over 82% of township level clinics, and receiving around 25,000 reports per day [31]. Wang et al. [28] suggest that the new system, and the reform of the mandatory reporting laws, have substantially changed the attitudes of providers: *“Before this, health-care providers and hospitals did not take disease reporting seriously”* (p1603).

IDRIMS can be viewed as a centrally managed relational database with direct links via a virtual private network (VPN) to healthcare providers, disease control and prevention agencies and health administration departments at all levels from township upwards. It was intended to bypass the previous hierarchical flow of information in which facilities would submit individual case reports to a local CDC, which would then prepare monthly summaries for analysis at the national level. It is linked to geographical information system software, which provides a real-time display, allowing users to identify case clusters in a given location which may indicate an outbreak of one of the monitored infectious diseases. By providing case-based as opposed to aggregated data, health officials can immediately analyse the characteristics (e.g. location, age, sex, occupation) of those suffering in that outbreak and, because the data reflects the situation within the previous 24 h, develop timely and appropriate plans for containment and treatment [28, 32].

The perceived success of the IDRIMS has led to the development of a number of disease-specific reporting initiatives extending the core database. These include Malaria [33, 34], Dengue [35], and Tuberculosis [36, 37]. In the case of Malaria, healthcare providers are encouraged to report both confirmed and suspected cases to the local CDC for rapid follow-up. This can be done using a mobile phone short message service (SMS) system, which was integrated into the IDRIMS following the Sichuan earthquake of 2008, when severe damage to both health facilities and communications infrastructure made their internet connection inoperable [38]. Detection of dengue outbreaks has made use of the Infectious Disease Automated-alert and Response System (IDARS) implemented in 2008, which conducts routine analysis of IDRIMS data using three anomaly detection methods to alert health officials to atypical disease patterns at county level, comparing each current seven day period to the corresponding period, and the preceding and following two week periods. The identification of an anomaly would result in a review of the data by an epidemiologist and a potential field visit to the site of suspected outbreak. CDCs at the county, prefecture, provincial and national levels participate in the system [35].

Perhaps the most sophisticated addition has been the TB Information Management System (TBIMS), which builds on IDRIMS data to track TB care from suspected cases to outcomes and includes substantial additional information relating to testing and treatment. An initial version was implemented in 2005 and substantially revised in 2009, with a drug-resistant TB module implemented in 2011 [37]. The system is used by all (around 3200) health facilities focusing on TB including TB dispensaries and designated hospitals at county, prefecture and provincial levels [36]. These facilities report confirmed cases to the TBIMS within 48 h and presumptive cases to the IDRIMS within 24 h. Other hospitals will refer patients seeking TB care to a TB dispensary or designated hospital and report the case to the IDRIMS. The referral unit will automatically receive notification of the case and can check if the patient did seek care, if the diagnosis was confirmed and if a positive diagnosis led to an entry in the TBIMS. Over the three years following introduction of the new system, the number of referred patients obtaining a diagnosis increased by more than 150%, “*mainly due to improved tracing of referred patients*” [37] (p939}. Around one million cases/suspects are entered into the system each year. A parallel paper-based system was run from 2005 to 2008 to assess the reliability of the new approach and implement data entry validation procedures which are said to have substantially reduced reporting errors [36]. Until the implementation of the new reporting system, the detection rate in China was only around 30% and multi-drug resistance was becoming an ever more serious concern. In 2005, the detection rate increased to an estimated 80% of cases [37] and by 2011 it had reached 90% [39].

### Challenges

As with almost all ICT innovations in the health sector, a familiar list of problems have been encountered by those implementing the IDRIMS. Facilities in remote areas may lack adequate computer facilities, for example being dependent on a single device, or internet services which are slow or unreliable. In 2007 it was estimated that around 5% of county and 29% of township facilities had to submit reports over the telephone to another institution with internet access [30], obviously increasing the risk of data entry errors. In spite of the investment in training, it was often difficult in some locations to find sufficient staff who were able to enter data reliably and efficiently. This gave rise to complaints about having to take on what was seen as the burden of long hours of data entry [31, 32]. In Jiangsu Province workers were given an incentive for each case of malaria reported to compensate for what was seen as an additional workload. This was reported to have led to a considerable increase in data quality [33]. Finally, the need for system maintenance and updating, given the enormous volume of data entered each day, requires a substantial team of programmers, computer operators and advisory staff with medical knowledge to address inevitable software errors and hardware malfunctions [31].

More generally, IDRIMS has another limitation that is very common in the area of digital health, usually described as a lack of interoperability [40]. The need, following the limitations exposed by the SARS outbreak, to undertake rapid reform of disease surveillance procedures could only be accomplished by creating a stand-alone vertical system. Comparison might be made to the long standing debate on the merits of horizontal versus vertical healthcare programs [41]. Under IDRIMS, hospitals are provided with standardised software by the CDC and instructed to designate a staff member to operate the system and enter reports. A doctor responsible for the patient with a notifiable disease is required to pass the IDRIMS data items, as specified in laws on the Prevention and Treatment of Infectious Diseases, to that individual. The same doctor must also provide the same data items and more to the Hospital Information System (HIS).

Because hospitals in China have a considerable degree of autonomy, they design or select HIS software entirely in line with what they see as their specific needs, greatly limiting the possibility for sharing information with other providers or public health agencies. *“Very few people engaging in HIS have recognized its potential usefulness in providing key information for public health in China, and the uses of HIS are limited to the administration of hospitals and their patients”* (p632) [32]. Similar issue arise with the TBIMS. This is fully interoperable with IDRIMS but not with the information systems used by the TB designated hospitals. Again, information must be entered separately into each system [36]. These limitations result not only in the inefficient use of staff time and delays in the reporting of information, but almost certainly lead to a loss in data quality and a lack of compatibility between different sources of information that can hamper the analysis and interpretation of health data.

The IDRIMS was established as a vertical project, driven by the political imperative to respond to the SARS crisis with a rapid and radical reform of the disease surveillance system. Prolonged negotiations on the design of an interoperable reporting system with facilities that had adopted a wide variety of individually designed information management systems was not a feasible option. However, twelve years after the initial intervention, there is a recognition that the need for interoperability remains, not only in terms of practical issues relating to potential improvements in data quality and timeliness, but also to allow the compilation of comprehensive patient-centred information on diagnosis, treatment and outcomes.

Central to this issue is the fact that there is no real incentive to generate information that might be extremely useful to those concerned with health policy or planning, but has no obvious relevance for the operational activities of a specific facility. One interesting example relates to the use of record identifiers. Each Chinese citizen is in possession of a Resident Identity Card (RIC), which includes a unique identification number. This number will be included on an individual’s IDRIMS entry which, in theory, should allow it to be linked to their facility medical records. However, while these records will typically include a field intended to capture the RIC number, in many cases it will not be completed because the facility has organised its data around an alternative, internal, reference code.

The hierarchical nature of the system is especially problematic in a country where healthcare is based on place of residence and where there is an internal migrant population of over 200 million. In the case of communicable diseases such as TB, which require an extended period of treatment, it is essential that patients who move from one locality to another can be easily traced and the process from diagnosis to treatment and outcome recorded. However, the data entered at county level by a TB dispensary or designated hospital is currently accessible only at higher levels, not to the corresponding facilities in other counties [36].

Another inevitable implication of establishing a surveillance system that focuses on a legally determined list of notifiable diseases, 37 in the case of IDRIMS, is that it may omit some important conditions. For example, extra-pulmonary TB, because of the low – but not negligible – risk of transmission, is not on the list, even in cases where it proves to be drug resistant. A more general problem is that any such surveillance system which is based to a very large extent on formally diagnosed case reports will almost certainly miss cases which arise in poorer rural areas. Many hospitals and clinics in these areas may lack the equipment and laboratory facilities to undertake robust diagnosis of some conditions, and village doctors, typically the first point of contact for patients, often lack training in the identification and diagnosis of some infectious diseases. This is a particularly important concern for diseases which have a higher incidence among those with poorer living conditions or unhealthy environments. There have been a number of recent proposals to establish an integrated surveillance system, using a ‘syndromic surveillance’ approach to complement traditional case report surveillance in rural China [42, 43].

## The Uganda case study

The mTrac component of eHMIS [44] was first piloted by the Foundation for Innovative New Diagnostics [45] in 2009/2010 in two Districts. In 2011, DFID approached the MoH, WHO and UNICEF with a highly ambitious proposal to scale the system nationally within one year. mTrac’s initial purpose at that time was to allow health facility staff to use their mobile phones to send weekly HMIS surveillance reports via SMS which included data on notifiable diseases, malaria treatment and stock levels of antimalarials to the District Local Government and the MoH. It also provided a toll-free ‘anonymous health service delivery’ hotline number which could be used by anyone to report via toll-free SMS messages on any health issue, including health worker absenteeism, fraud, extortion, assault and theft of medicines [45, 46]. The underlying technology was based on RapidSMS, an open source web framework developed by the UNICEF Innovation Unit, which provides a tool for rapidly developing and deploying SMS based systems at scale [47].

With the mHealth moratorium in place, mTrac was one of only a handful of initiatives approved by the MoH to scale-up. It was initiated in some 1000 facilities in 28 districts by March 2012 and by March 2013 was scaled up to every government health facilities in all 112 districts. By June 2015, mTrac had been extended to include most non-government and private health facilities, totalling approximately 4400. Investments in creating an enabling environment – including updating national policies, capacity building, software development, provision of computers and modems to districts, establishment of the hotline and staff training required an initial investment, mainly by international donors, of some US$4.5 million. The MoH has assumed responsibility for some of the comparatively limited recurrent costs, primarily relating to internet connections and maintaining the software, though not the cost of the SMS service [48].

Each week, facility health workers use their own mobile phones to submit short SMS messages, each of which starts with a keyword relating to a distinct element of the standard HMIS form 033b [49] (p551). These messages are received at the MoH Resource Centre in Kampala and feed automatically into a central web-based database, where District Health staff can immediately access the data online for review, verification and, where necessary, correction by the relevant District Health Team (DHT) and selected national stakeholders. Once validated, the data is automatically fed into the District Health Information System (DHIS2), the central database used for the analysis of national level health services data, where both aggregated and disaggregated data can be analysed using pivot tables, graphs and a Geographical Information System (GIS). Summary reports are sent by the MoH Resource Centre to key action centres, including the Epidemiology Surveillance Division, the Pharmacy Division, the National Malaria Control Programme and collaborating health development partners [50].

The mTrac system also initiates real-time SMS ‘alerts’ to warn district and national stakeholders when certain actionable data is reported, such as cases of viral haemorrhagic fever and cholera, to provoke immediate and appropriate interventions. Similar procedures were initially introduced for the community-based volunteers who constitute the village health teams (VHTs). They also submitted weekly data, in this case from MoH form 097, the VHT register [49] (p509), including stocks of ACT and Amoxycillin [44]. However, the national medicine supply chain was unable to react and respond to increasing cases of stock-outs at the community level, and this component of mTrac was temporarily put on hold.

mTrac is the primary source of weekly HMIS data for the DHIS2. District biostatisticians analyse the data collected through mTrac using DHIS2 to review trends in facility reporting performance, disease incidence rates and drug stock levels. Analysis at the national level is undertaken by the MoH Resource Centre in collaboration with other divisions and agencies. Following the introduction of mTrac, stock-outs of ACTs decreased from 25% to 14% over an 18-month period [51]. Reporting rates for the surveillance form 033b, which were around 50% in the first week of 2015, reached 68% by December 2015, the highest proportion for the public sector since the form was introduced. In addition, data was gathered from over 2000 non-government health facilities [52].

A designated team of officials at the MoH, who review each message and pass it to the appropriate agency, initially handles the widely publicised toll-free hotline phone number. About 90% of reports are sent back to the Districts for action, but in some cases – particularly those of a criminal nature – they are immediately forwarded to national level organisations such as the State House’s Health Monitoring Unit. Each report requires action centres to indicate what response was taken via the mTrac dashboard. During 2014/15, a total of 9214 SMS texts were received over the hotline, with some 40% of these designated as “actionable”. Of those addressed at the district level, some 64% were successfully resolved within a two-week period, while the Health Monitoring Unit took action on 93% of more serious complaints. This included the recovery of over 60 million Ugandan Schillings (around US$8000) worth of medicines stolen from Mulago National Hospital and the prosecution of numerous cases of extortion and negligence (some of which led to preventable maternal and neo-natal deaths).

From its initial implementation, the need for mTrac to become fully integrated into the overall government health information system (HIS) has always been a priority. This began with designing the system using existing MoH HMIS reporting forms. A crucial next step was integration with DHIS2, which is used by all Districts in Uganda. The initial version of this web-based, open source software was developed in South Africa in 1996, with support from the University of Oslo, and gradually implemented in all health districts in South Africa country by 2001 [53]. Details of this challenging process are provided in Braa and Hedberg [54]. The software is currently in use in 47 countries, with further development being undertaken in Vietnam, India, Tanzania, Norway, Ireland and the United States. It is supported by NORAD, the Research Council of Norway, PEPFAR and The Global Fund [55]. It was customised to align with the reporting requirements of the MoH in Uganda by the University of Oslo and identified as the only approved national HMIS system in January 2011. Following an initial pilot, it was scaled up to all 112 districts by July 2012. An evaluation by Kibaru et al. [56] found dramatic improvements in routine reporting, with completeness increasing from 36% to 85% for monthly outpatient reports and 21% to 56% for inpatient reports by 2012/13. Similar gains were seen in terms of timeliness of reporting.

As part of the MoH’s push towards a fully interoperable national HIS, UNICEF and the University of Oslo has created a centralized Health Facility Registry, using emerging standards, application protocol interfaces (API’s) and best practices coming out of the global OpenHIE community. A similar process is underway to establish a centralized Health Care Provider registry, with support from Jembi Health Systems, a South African NGO [57]. To emphasise the aim of full integration of mTrac and DHIS2, in 2015 the MoH rebranded these tools as the “electronic Health Management Information System” (eHMIS), and integrated the technical working groups. The MoH’s next goal is to move services to the patient and community level, and introduce electronic medical records, which they aim to scale up by 2020.

### Challenges

As might be expected, there have been issues relating to infrastructure issues in a number of districts. These relate to both failures in the electricity supply and limited internet bandwidth, which hamper the work of the DHTs. Kiberu et al. [56] also note that the MoH itself was routinely subject to electricity black-outs, which hindered access to the central server. While it appears that mTrac has made routine reporting more efficient, and as a result more attractive, to health workers [48], the data still needs to be compiled from the various source documents. Additionally, private sector facilities have performed significantly below the national average, as there is little motivation or leverage to compel timely reporting. Furthermore, while many Districts are avid consumers of the data – as seen by Districts such as Yumbe that are now reporting close to zero preventable intra-district drug stock-outs, there remain significant gaps in data utilization and accountability for response across the country. UNICEF, with partners, is prioritizing support to the government around this issue in 2016 through a national score card accountability strategy. Given that the initiative is still at a relatively early stage, it will be interesting to assess if the enthusiasm of MoH, District health staff, and health workers can be maintained, or if the training and encouragement provided at this early stage, a substantial proportion of the start-up costs, will need to be reinforced on a regular basis or additional incentives devised.

mTrac was built around existing MoH reporting formats and, in line with a national eHealth policy, has recently been formally absorbed into the eHMIS programme. It is now required to synchronise facility data with DHIS2, health care provider data with iHRIS (a database used to capture data about health worker training, deployment, registration and licensure), and routinely feed data into DHIS2 for analysis. The need to manage multiple systems, and overcome issues arising from potentially worthwhile upgrades that might break compatibility with existing systems, has been challenging. Each modification to a national HMIS necessarily involves a substantial new round of training and the distribution of training and operational materials. Typically this has resulted in a temporary reduction in the quantity and quality of submitted reports. Table 1 shows the impact of changes introduced in the first quarter of financial year 2015/16.

**Table 1:** Reporting rates for 112 districts for financial year 2015/16

Reporting Rate	No. of Districts		
	Q1	Q2	Q3
100	4	18	24
80–99	16	37	59
79–50	53	45	26
<50	39	12	3

Source: Uganda Ministry of Health. eHMIS Annual Donor Report 2016

This reflects a more general problem that most ministries are not equipped with the institutional or individual expertise required to undertake what has become a very substantial programme of work. To address this issue, those promoting the eHMIS have successfully advocated for a restructuring of a major unit at the MoH into a Division of Health Information, which will include proper staffing with appropriate skills.

One well known effect of improving health information systems is a rise in the reported incidence of common diseases, as was described above in the case of TB in China. This also seems to have been one outcome of accessing more reliable and disaggregated data, as well as providing a simple means for community members to report on concerns relating to health services. The Health Monitoring Unit [58] notes a steady increase in complaints over time. While many relate to issues, for example drug stock-outs, that can be relatively easily resolved, report categories include ‘Theft, Forgery, Extortion and Harassment’, which suggest that resolution may require extensive investigation and possibly negotiation with local staff or communities, creating additional workload on local and national officials. This is critical because, as with all such systems, it will only continue to be used over the long run if it is perceived that complaints are dealt with in an effective, timely manner.

## Discussion

Interoperability has been identified as the key to maximising the healthcare benefits of recent development in ICTs [59, 60]. However, while there is general agreement that each component of an HMIS should ideally aim to allow data exchange with all other components, the transaction costs associated with this goal, particularly in terms of the time required for implementation, should not be underestimated [61]. Those who implement eHealth innovations are typically convinced of the potential value of those innovations and reluctant to delay their implementation in order to meet what they may see as the unreasonable reluctance of other stakeholders to accept new ways of working. On the other hand those stakeholders may have substantial investments in terms of expertise and experience in using the existing systems and feel justified in demanding substantial evidence of the potential net benefits of the proposed innovation before they are willing to accept it. The difficult and prolonged negotiations involved in the introduction of the original version of the DHIS in South Africa provides a very interesting example of this process [54, 62].

In the above examples, alternative strategies have been adopted. For those implementing mTrac, conformity with existing MoH reporting systems was seen as essential from the outset in order to win the support of key public sector stakeholders. Improvement of those systems could be delayed until mTrac was firmly established. In China, the existing systems were seen to have proved not fit for purpose, requiring that reform took priority over interoperability. It would seem that the process of achieving this second goal is proving a prolonged and difficult task.

In both countries, data quality is dependent on the ability *and* willingness both of those managing the new systems and those designated as the providers of primary data. The limited expertise available at ministerial level in Uganda has been mentioned above. At a grass roots facility level even the basic skills required to provide data may in some cases be lacking. A recent study of staff at this level in South Africa, which has been using DHIS2 since 1999, reports that “*findings suggest that 64% of the respondents have poor numerical skills and limited statistical and data quality checking skills … only 22% actually displayed competence above 50%. Personnel appear to be reasonably motivated but there is considerable deficiency in their competency to interpret and use data*” [63] (p789). This would suggest that training and supervision of those entering data will be key determinants of the quality of any surveillance system, a similar finding to that from a recent systematic review [64]. However, it seems probable that the status accorded to the activities of these individuals by the institution within which they work may strongly influence the level of support provided.

In China, the main issue appears to be the lack of engagement by facility staff with the IDRIMS and TBIMS. As indicated above, facilities which have a high degree of autonomy have typically implemented information systems designed primarily to meet their internal needs, while allowing them to satisfy the limited reporting requirements of government and insurance agencies. Even the legal requirement for facilities to provide data on patients diagnosed with a notifiable disease does not seem to provide a sufficient incentive, probably because there is little possibility that they will be penalised if they fail to comply. Given that financial incentives appear to be the primary influence on provider behaviour and that reimbursement of fees by insurance schemes constitutes a major component of facility income, one possible way forward might be to make reimbursement for a patient with a notifiable disease contingent on the insurance agency being able to locate that patient on the relevant database.

Those involved in the Uganda exercise are very aware that while greater transparency and accountability through increased access to reliable data may be widely regarded as an important driver of improvements in health services, they can also be perceived as very threatening by some key stakeholders. To take an extreme example, the anonymous reporting system which was introduced as part of mTrac, combined with improved logistical and financial data, is exposing corrupt behaviour, sometimes at a level that may seriously compromise service delivery, for example through the misappropriation of essential drugs. In China, where facilities have typically been encouraged to act in a manner similar to that of private enterprises in a free market, with an emphasis on self-reliance and at least to some extent a competitive attitude to other providers, the potential for greater scrutiny by outside agencies, possibly with the potential for increased or more effective regulation, will almost inevitably be seen as presenting a substantial risk to their current status.

More generally, improved access to timely data by agencies that have the incentive and capacity to analyse that data will almost certainly expose long standing examples of poor practice and inefficiency. Even the most honest and committed providers, managers and administrators may reasonably feel threatened if they perceive that new information systems may be used to allocate blame rather than to seek new ways of working that improve their situation. This requires a careful process of change management, addressing the concerns of stakeholders where possible, identifying champions who can explain the potential benefits and address the perceived risks of new approaches to their colleagues, and, especially in areas of accountability, working incrementally rather than demanding radical reforms.

Finally, in both examples, it is clear that the key to driving up data quality is the utilisation of the data by the various stakeholders [65]. In Uganda, it has become clear that those districts with over 90% reporting rates are actively using the data to improve health outcomes, while in others low reporting rates are reflected in the limited role which the data play in operational activities. In many of these district, for example, drug stock-outs are common even though the information required to take effective action is available. In China, the IDRIMS and related TBIMS are actively used by the CDC in advancing their work on disease surveillance and prevention and they therefore make great efforts to ensure the reliability of the data collected. However, that data appears to remain largely irrelevant to the activities of the facilities from which it is gathered, who have therefore shown limited interest in plans for integration with the hospital information systems.

## Conclusions

Both examples, stress the importance of recognising the political, social and historical context within which information systems have to function. Implementers need to focus as much on the perceptions, attitudes and needs of stakeholders as on the technology. It may also be useful to distinguish between factors which may influence engagement at an institutional level, for example linking provision of resources to quality data delivery and emphasising a ‘lesson-learning’ rather than a ‘blame-allocation’ ethos, and those aimed at supporting and supervising individuals within those institutions. Finally, we would suggest that designing interoperability into systems at the outset, rather than assuming that this can be achieved at some point in the future, may prove far easier in the longer term.
